# Development of an index system for the scientific literacy of medical staff: a modified Delphi study in China

**DOI:** 10.1186/s12909-024-05350-0

**Published:** 2024-04-10

**Authors:** Shuyu Liang, Ziyan Zhai, Xingmiao Feng, Xiaozhi Sun, Jingxuan Jiao, Yuan Gao, Kai Meng

**Affiliations:** 1 Aerospace Center Hospital, No. 15 Yuquan Road, Haidian District, Beijing, 100049 China; 2 School of Public Health, Capital Medical University, No.10 Xitoutiao, Youanmenwai Street, Fengtai District, Beijing, 100069 China; 3 Beijing Tiantan Hospital, Capital Medical University, No. 119 South Fourth Ring West Road, Fengtai District, Beijing, 100070, China

**Keywords:** Medical staff, Scientific literacy, Evaluation indicators, Delphi

## Abstract

**Background:**

Scientific research activity in hospitals is important for promoting the development of clinical medicine, and the scientific literacy of medical staff plays an important role in improving the quality and competitiveness of hospital research. To date, no index system applicable to the scientific literacy of medical staff in China has been developed that can effectively evaluate and guide scientific literacy. This study aimed to establish an index system for the scientific literacy of medical staff in China and provide a reference for improving the evaluation of this system.

**Methods:**

In this study, a preliminary indicator pool for the scientific literacy of medical staff was constructed through the nominal group technique (*n* = 16) with medical staff. Then, two rounds of Delphi expert consultation surveys (*n* = 20) were conducted with clinicians, and the indicators were screened, revised and supplemented using the boundary value method and expert opinions. Next, the hierarchical analysis method was utilized to determine the weights of the indicators and ultimately establish a scientific literacy indicator system for medical staff.

**Results:**

Following expert opinion, the index system for the scientific literacy of medical staff featuring 2 first-level indicators, 9 second-level indicators, and 38 third-level indicators was ultimately established, and the weights of the indicators were calculated. The two first-level indicators were research literacy and research ability, and the second-level indicators were research attitude (0.375), ability to identify problems (0.2038), basic literacy (0.1250), ability to implement projects (0.0843), research output capacity (0.0747), professional capacity (0.0735), data-processing capacity (0.0239), thesis-writing skills (0.0217), and ability to use literature (0.0181).

**Conclusions:**

This study constructed a comprehensive scientific literacy index system that can assess medical staff's scientific literacy and serve as a reference for evaluating and improving their scientific literacy.

**Supplementary Information:**

The online version contains supplementary material available at 10.1186/s12909-024-05350-0.

## Background

Due to the accelerated aging of the population and the growing global demand for healthcare in the wake of epidemics, there is an urgent need for medicine to provide greater support and protection. Medical scientific research is a critical element in promoting medical science and technological innovation, as well as improving clinical diagnosis and treatment techniques. It is the main driving force for the development of healthcare [1].

Medical personnel are highly compatible with clinical research. Due to their close interaction with patients, medical staff are better equipped to identify pertinent clinical research issues and actually implement clinical research projects [2]. Countries have created favorable conditions for the research and development of medical personnel by providing financial support, developing policies, and offering training courses [3, 4]. However, some clinical studies have shown that the ability of most medical staff does not match current health needs and cannot meet the challenges posed by the twenty-first century [5]. It is clear that highly skilled professionals with scientific literacy are essential for national and social development [6]. Given the importance of scientific research in countries and hospitals, it is crucial to determine the level of scientific research literacy that medical personnel should possess and how to train them to acquire the necessary scientific research skills. These issues have significant practical implications.

Scientific literacy refers to an individual's ability to engage in science-related activities [7]. Some scholars suggest that the scientific literacy of medical personnel encompasses the fundamental qualities required for scientific research work, encompassing three facets: academic moral accomplishment, scientific research theory accomplishment, and scientific research ability accomplishment [8]. The existing research has focused primarily on the research capabilities of medical staff. According to Rillero, problem-solving skills, critical thinking, communication skills, and the ability to interpret data are the four core components of scientific literacy [9]. The ability to perform scientific research in nursing encompasses a range of abilities, including identifying problems, conducting literature reviews, designing and conducting scientific research, practicing scientific research, processing data, and writing papers [10]. Moule and Goodman proposed a framework of skills that research-literate nurses should possess, such as critical thinking capacity, analytical skills, searching skills, research critique skills, the ability to read and critically appraise research, and an awareness of ethical issues [11]. Several researchers have developed self-evaluation questionnaires to assess young researchers' scientific research and innovative abilities in the context of university-affiliated hospitals (UHAs) [12]. The relevant indicators include sensitivity to problems, sensitivity to cutting-edge knowledge, critical thinking, and other aspects. While these indicators cover many factors, they do not consider the issue of scientific research integrity in the medical field. The lack of detailed and targeted indicators, such as clinical resource collection ability and interdisciplinary cooperation ability, hinders the effective measurement of the current status of scientific literacy among medical staff [12]. In conclusion, the current research on the evaluation indicators of scientific literacy among medical personnel is incomplete, overlooking crucial humanistic characteristics, attitudes, and other moral literacy factors. Therefore, there is an urgent need to establish a comprehensive and systematic evaluation index to effectively assess the scientific literacy of medical staff.

Therefore, this study utilized a literature search and nominal group technique to screen the initial evaluation index and subsequently constructed an evaluation index system for medical staff's scientific research literacy utilizing the Delphi method. This index system would serve as a valuable tool for hospital managers, aiding them in the selection, evaluation, and training of scientific research talent. Additionally, this approach would enable medical personnel to identify their own areas of weakness and implement targeted improvement strategies.

## Methods

### Patient and public involvement

Patients and the public were not involved in this research.

### Study design and participants

In this study, an initial evaluation index system was developed through a literature review and nominal group technique. Subsequently, a more comprehensive and scientific index system was constructed by combining qualitative and quantitative analysis utilizing the Delphi method to consult with experts. Finally, the hierarchical analysis method and the percentage weight method were employed to empower the index system.

**Fig. 1:**
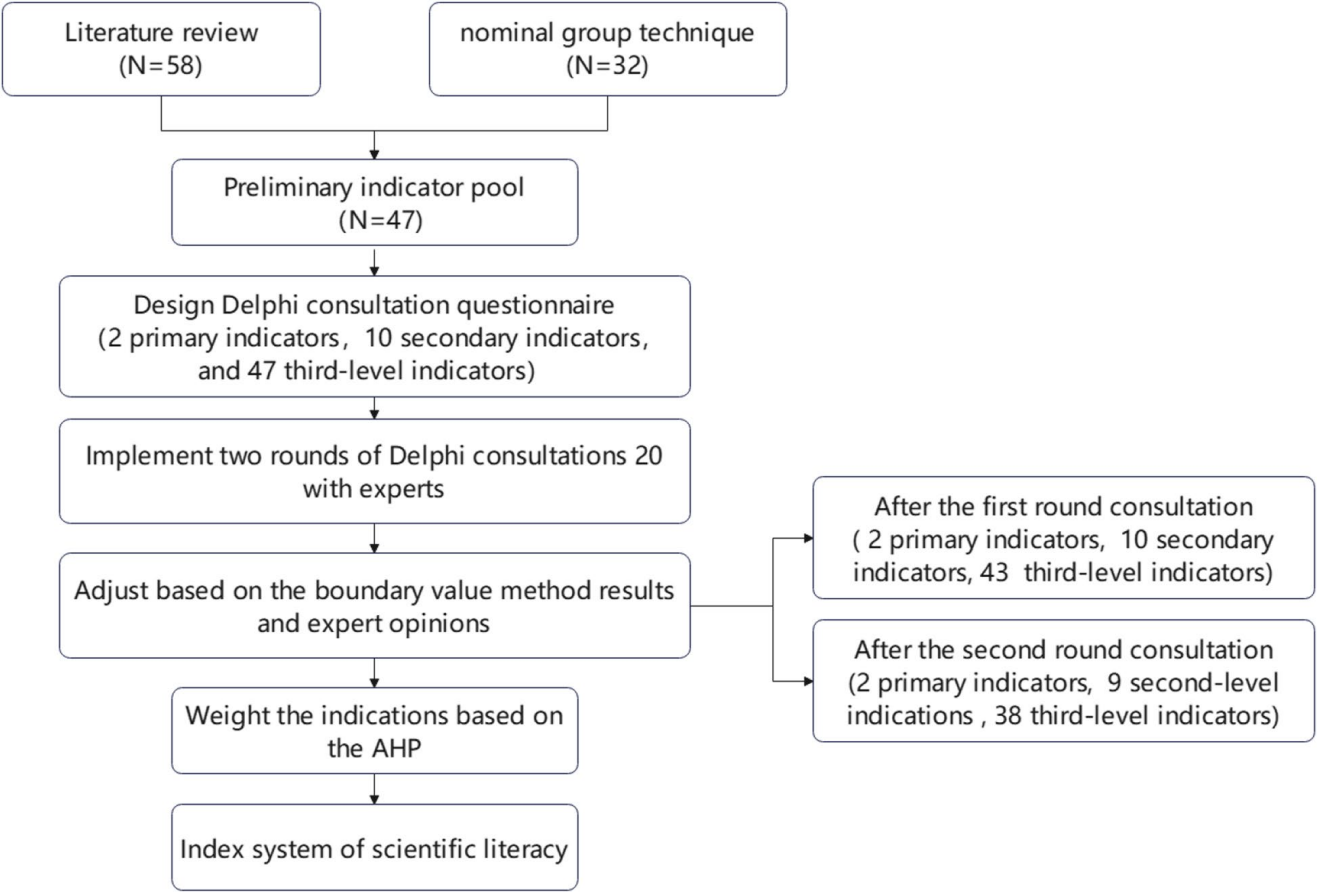
Study design. AHP, analytic hierarchy process

The program used for this study is shown in Fig. 1.

### Establishing the preliminary indicator pool

#### Search process

A literature search was performed in the China National Knowledge Infrastructure (CNKI), WanFang, PubMed, Web of Science and Scopus databases to collect the initial evaluation indicators. The time span ranged from the establishment of the database to July 2022. We used a combination of several MeSH terms in our searches:(("Medical Staff"[Mesh] OR "Nurses"[Mesh] OR "Physicians"[Mesh])) AND (("Literacy"[Mesh]) OR "Aptitude"[Mesh]). We also used several Title/Abstract searches, including keywords such as: Evaluation, scientific literacy, research ability.

The inclusion criteria were as follows: (1)The subjects were nurses, medicial staff and other personnel engaged in the medical industry; (2) Explore topics related to scientific literacy, such as research ability, and literature that can clarify the structure or dependency between indicators of scientific literacy; (3) Select articles published in countries such as China, the United States, the United Kingdom, Australia and Canada; (4) Research published in English or Chinese is considered to be eligible. The exclusion criteria are as follows: (1) indicators not applicable to medical staff; (2) Conference abstracts, case reports or review papers; (3) Articles with repeated descriptions; (4) There are no full-text articles or grey literature. A total of 78 articles were retrieved and 60 were retained after screening according to inclusion and exclusion criteria.

The research was conducted by two graduate students and two undergraduate students who participated in the literature search and screening. The entire research process was supervised and guided by one professor. All five members were from the fields of social medicine and health management. The professor was engaged in hospital management and health policy research for many years.

#### Nominal group technique

The nominal group technique was introduced at Hospital H in Beijing in July 2022. This hospital, with over 2,500 beds and 3,000 doctors, is a leading comprehensive medical center also known for its educational and research achievements, including numerous national research projects and awards.

The interview questions were based on the research question: What research literacy should medical staff have? 16 clinicians and nurses from Hospital H were divided into 2 equal groups and asked to provide their opinions on important aspects of research literacy based on their positions and experiences. Once all participants had shared their thoughts, similar responses were merged and polished. If anyone had further inputs after this, a second round of interviews was held until no new inputs were given. The entire meeting, including both rounds, was documented by researchers with audio recordings on a tape recorder.

#### Scientific literacy dimensions

Based on the search process, the research group extracted 58 tertiary indicators. To ensure the practicality and comprehensiveness of the indicators, the Nominal group technique was used on the basis of the literature search. Panelists summarized the entries shown in the interviews and merged similar content to obtain 32 third-level indicators. The indicators obtained from the literature search were compared. Several indicators with similar meanings, such as capture information ability, language expression ability, communication ability, and scientific research integrity, were merged. Additionally, the indicators obtained from the literature search, such as scientific research ethics, database use ability, feasibility and analysis ability, were added to the 15 indicators. A total of 47 third-level indicators were identified.

Fengling Dai and colleagues developed an innovation ability index system with six dimensions covering problem discovery, information retrieval, research design, practice, data analysis, and report writing, which represents the whole of innovative activity. Additionally, the system includes an innovation spirit index focusing on motivation, thinking, emotion, and will, reflecting the core of the innovation process in terms of competence [13]. Liao et al. evaluated the following five dimensions in their study on scientific research competence: literature processing, experimental manipulation, statistical analysis, manuscript production, and innovative project design [14]. Mohan claimed that scientific literacy consists of four core components: problem solving, critical thinking, communication skills, and the ability to interpret data [15].

This study structured scientific literacy into 2 primary indicators (research literacy and research competence) and 9 secondary indicators (basic qualifications, research ethics, research attitude, problem identification, literature use, professional capacity, subject implementation, data processing, thesis writing, and research output).

### Using the Delphi method to develop an index system

#### Expert selection

This study used the Delphi method to distribute expert consultation questionnaires online, allowing experts to exchange opinions anonymously to ensure that the findings were more desirable and scientific. No fixed number of experts is required for a Delphi study, but the more experts involved, the more stable the results will be [16]; this method generally includes 15 to 50 experts [17]. We selected clinicians from several tertiary hospitals in the Beijing area to serve as Delphi study consultants based on the following inclusion criteria: (1) they had a title of senior associate or above; (2) they had more than 10 years of work experience in the field of clinical scientific research, and (3) they were presiding over national scientific research projects. The exclusion criteria were as follows: (1) full-time scientific researchers, and (2) personnel in hospitals who were engaged only in management. To ensure that the selected experts were representative, this study selected 20 experts from 14 tertiary hospitals affiliated with Capital Medical University, Peking University, the Chinese Academy of Medical Sciences and the China Academy of Traditional Chinese Medicine according to the inclusion criteria; the hospitals featured an average of 1,231 beds each, and 9 hospitals were included among the 77 hospitals in the domestic comprehensive hospital ranking (Fudan Hospital Management Institute ranking). The experts represented various specialties and roles from different hospitals, including cardiology, neurosurgery, neurology, ear and throat surgery, head and neck surgery, radiology, imaging, infection, vascular interventional oncology, pediatrics, general practice, hematology, stomatology, nephrology, urology, and other related fields. This diverse group included physicians, nurses, managers, and vice presidents. The selected experts had extensive clinical experience, achieved numerous scientific research accomplishments and possessed profound knowledge and experience in clinical scientific research. This ensured the reliability of the consultation outcomes.

#### Design of the expert consultation questionnaire

The Delphi survey for experts included sections on their background, familiarity with the indicator system, system evaluation, and opinions. Experts rated indicators on importance, feasibility, and sensitivity using a 1–10 scale and their own familiarity with the indicators on a 1–5 scale. They also scored their judgment basis and impact on a 1–3 scale, considering theoretical analysis, work experience, peer understanding, and intuition. Two rounds of Delphi surveys were carried out via email with 20 experts to evaluate and suggest changes to the indicators. Statistical coefficients were calculated to validate the Delphi process. Feedback from the first round led to modifications and the inclusion of an AHP questionnaire for the second round. After the second round, indicators deemed less important were removed, and expert discussion finalized the indicator weights based on their relative importance scores. This resulted in the development of an index system for medical staff scientific literacy. The questionnaire is included in Additional file 1 (first round) and Additional file 2 (second round).

#### Using the boundary value method to screen the indicators

In this study, the boundary value method was utilized to screen the indicators of medical staff's scientific literacy, and the importance, feasibility, and sensitivity of each indicator were measured using the frequency of perfect scores, the arithmetic mean, and the coefficient of variation, respectively. When calculating the frequency of perfect scores and arithmetic means, the boundary value was set as "mean-SD," and indicators with scores higher than this value were retained. When calculating the coefficient of variation, the cutoff value was set to "mean + SD," and indicators with values below this threshold were retained.

The principles of indicator screening are as follows:

1. To evaluate the importance of the indicators, if none of the boundary values of the three statistics met the requirements, the indicators were deleted.
2. If an indicator has two aspects, importance, feasibility, or sensitivity, and each aspect has two or more boundary values that do not meet the requirements, then the indicator is deleted.
3. If all three boundary values for an indicator meet the requirements, the research group discusses the modification feedback from the experts and determines whether the indicator should be used.

The results of the two rounds of boundary values are shown in Table 1.

**Table 1:** Results of the two rounds of the boundary value method

Round	Dimension	Importance			Feasibility			Sensitivity		
		**M**	**S**	**BD**	**M**	**S**	**BD**	**M**	**S**	**BD**
First	Full score frequency	0.42	0.14	0.27	0.25	0.10	0.14	0.24	0.80	0.16
	Arithmetic mean	8.74	0.47	8.27	7.68	0.56	7.12	7.64	0.50	7.14
	Coefficient of variation	0.16	0.04	0.20	0.26	0.06	0.32	0.26	0.07	0.32
Second	Full score frequency	0.64	0.13	0.50	0.33	0.12	0.21	0.33	0.12	0.21
	Arithmetic mean	4.56	0.19	4.37	3.91	0.27	3.64	3.88	0.23	3.65
	Coefficient of variation	0.14	0.04	0.17	0.25	0.05	0.29	0.25	0.05	0.30

M represents the arithmetic mean, S represents the SD and BD represents the boundary value

#### Using the AHP to assign weights

After the second round of Delphi expert consultations, the analytic hierarchy process (AHP) was used to determine the weights of the two first-level indicators and the nine second-level indicators. The weights of the 37 third-level indicators were subsequently calculated via the percentage weight method. The AHP, developed by Saaty in the 1980s, is used to determine the priority and importance of elements constituting the decision-making hierarchy. It is based on multicriteria decision-making (MCDM) and determines the importance of decision-makers' judgments based on weights derived from pairwise comparisons between elements. In the AHP, pairwise comparisons are based on a comparative evaluation in which each element's weight in the lower tier is compared with that of other lower elements based on the element in the upper tier [18].

AHP analysis involves the following steps:

- Step 1: Establish a final goal and list related elements to construct a hierarchy based on interrelated criteria.
- Step 2: Perform a pairwise comparison for each layer to compare the weights of each element. Using a score from 1 to 9, which is the basic scale of the AHP, each pair is compared according to the expert’s judgment, and the importance is judged [19, 20].

Yaahp software was employed to analyze data by creating a judgment matrix based on the experts' scores and hierarchical model. The index system weights were obtained by combining the experts' scores. The percentage weight method used experts' importance ratings from the second round to calculate weights, ranking indicators by importance, calculating their scores based on frequency of ranking, and determining weighting coefficients by dividing these scores by the total of all third-level indicators' scores. The third-level indicator weighting coefficients were then calculated by multiplying the coefficients [21].

### Data analysis

#### Expert positivity coefficient

The expert positivity coefficient is indicated by the effective recovery rate of the expert consultation questionnaire, which represents the level of expert positivity toward this consultation and determines the credibility and scientific validity of the questionnaire results. Generally, a questionnaire with an effective recovery rate of 70% is considered very good [22].

In this study, 20 questionnaires were distributed in both rounds of Delphi expert counseling, and all 20 were effectively recovered, resulting in a 100% effective recovery rate. Consequently, the experts provided positive feedback on the Delphi counseling.

#### Expert authority coefficient (CR)

The expert authority coefficient (Cr) is the arithmetic mean of the judgment coefficient (Ca) and the familiarity coefficient (Cs), namely, Cr = $\frac{({\text{Ca}}+{\text{Cs}})}{2}$. The higher the degree of expert authority is, the greater the predictive accuracy of the indicator. A Cr ≥ 0.70 was considered to indicate an acceptable level of confidence [23]. Ca represents the basis on which the expert makes a judgment about the scenario in question, while Cs represents the expert's familiarity with the relevant problem [24].

Ca is calculated on the basis of experts' judgments of each indicator and the magnitude of its influence. In this study, experts used "practical experience (0.4), "theoretical analysis (0.3), "domestic and foreign peers (0.2)" and "intuition (0.1)" as the basis for judgment and assigned points according to the influence of each basis for judgment on the experts' judgment. Ca = 1 when the basis for judgment has a large influence on the experts, and Ca = 0.5 when the influence of the experts' judgment is at a medium level. When no influence on expert judgment was evident, Ca = 0 [25] (Table 2).

**Table 2:** Judgment basis and the degree of influence

Judgment basis	Degree of influence		
	**Low (0)**	**Medium (0.5)**	**High (1)**
Practical experience (0.4)	0	0.2	0.4
Theoretical analysis (0.3)	0	0.15	0.3
Domestic and foreign peers (0.2)	0	0.1	0.2
Intuition (0.1)	0	0.05	0.1
Total	0	0.5	1

Cs refers to the degree to which the expert was familiar with the question. This study used the Likert scale method to score experts’ familiarity with the question on a scale ranging from 0 to 1 (1 = very familiar, 0.75 = more familiar, 0.5 = moderately familiar, 0.25 = less familiar, 0 = unfamiliar). The familiarity coefficient for each expert (the average familiarity for each indicator) was calculated. The average familiarity coefficient was subsequently computed [26].

The Cr value of the primary indicator in this study was 0.83, and the Cr value of the secondary indicator was 0.82 (> 0.7); hence, the results of the expert consultation were credible and accurate, as shown in Table 3.

**Table 3:** Expert authority coefficients

Dimension	Indicator name	Ca	Cs	Cr
Primary indicator	Research literacy	0.69	0.96	0.83
	Research ability	0.71	0.96	0.84
	**M**	0.70	0.96	0.83
Secondary indicator	Basic qualification	0.65	0.95	0.80
	Research ethics	0.70	0.96	0.83
	Research attitude	0.64	0.96	0.80
	Ability to identify problems	0.74	0.98	0.86
	Ability to use literature	0.77	0.98	0.87
	Professional capacity	0.70	0.96	0.83
	Subject implementation capacity	0.65	0.93	0.79
	Data-processing capacity	0.64	0.90	0.77
	Thesis-writing skills	0.71	0.94	0.82
	Research output capacity	0.70	0.88	0.79
	**M**	0.69	0.94	0.82

Ca represents the judgment coefficient, Cs represents the degree of familiarity and Cr represents the degree of authority

The degree of expert coordination is an important indicator used to judge the consistency among various experts regarding indicator scores. This study used the Kendall W coordination coefficient test to determine the degree of expert coordination. A higher Kendall W coefficient indicates a greater degree of expert coordination and greater consistency in expert opinion, and *P* < *0.05* indicates that the difference is significant [26]. The results of the three-dimensional harmonization coefficient test for each indicator in the two rounds of the expert consultation questionnaire were valid (*p* < *0.01*), emphasizing the consistency of the experts' scores. The values of the Kendall W coordination coefficients for both rounds are shown in Table 4.

**Table 4:** Kendall’s W concordance coefficient test results

	**First round**			**Second round**		
	**Importance**	**Feasibility**	**Sensitivity**	**Importance**	**Feasibility**	**Sensitivity**
Kw	0.195	0.118	0.098	0.112	0.108	0.082
*χ*^*2*^	179.138	108.619	89.816	93.845	90.429	69.005
*P* value	< 0.001	< 0.001	< 0.001	< 0.001	< 0.001	0.005

## Results

### Basic information regarding the participants

The 20 Delphi experts who participated in this study were predominantly male (80.0%) rather than female (20.0%). In addition, the participants’ ages were mainly concentrated in the range of 41–50 years old (60.0%). The majority of the experts were doctors by profession (85.0%), and their education and titles were mainly doctoral degree (90.0%) and full senior level (17.0%). The experts also exhibited high academic achievement in their respective fields and had many years of working experience, with the majority having between 21 and 25 years of experience (40.0%) (Table 5).

**Table 5:** Characteristics of the Delphi participants

Participants’ information	N	%
**Gender**		
Male	16	80.0
Female	4	20.0
**Age (years)**		
31–40	2	10.0
41–50	12	60.0
51–60	6	30.0
**Occupation**		
Doctor	17	85.0
Both hospital manager and doctor	3	15.0
**Education**		
Master's	2	10.0
PhD	18	90.0
**Professional title**		
Vice Senior	3	15.0
Full Senior	17	85.0
**Years worked**		
11–15	3	15.0
16–20	2	10.0
21–25	8	40.0
26–30	5	25.0
> 30	2	10.0

### Index screening

The boundary value method was applied to eliminate indicators, leading to the removal of 6 third-level indicators in the first round. One of these, the ability to use statistical software, was associated with a more significant second-level indicator involving data processing, which was kept after expert review. Six indicators were merged into three indicators due to duplication, and 5 third-level indicators were added, resulting in 2 primary indicators, 10 secondary indicators, and 43 third-level indicators.

In the second round of Delphi expert consultation, 5 third-level indicators were deleted, as shown in Additional file 3, and only one third-level indicator, "Scientific spirit", remained under the secondary indicator "research attitude". The secondary indicator "Research attitude" was combined with "Research ethics" and the third-level indicator "Scientific spirit" was also considered part of "Research ethics". After expert discussion, these were merged into a new secondary indicator "Research attitude" with three third-level indicators: "Research ethics", "Research integrity", and "Scientific spirit". The final index system included two primary indicators, nine secondary indicators, and thirty-eight third-level indicators, as shown in Additional File 3.

### Final index system with weights

The weights of the two primary indexes, research literacy and research ability, were equal. This was determined using the hierarchical analysis method and the percentage weight method based on the results of the second round of Delphi expert consultation (Table 6). The primary indicator of research literacy encompasses the fundamental qualities and attitudes medical staff develop over time, including basic qualifications and approach to research. The primary indicator of research ability refers to medical professionals' capacity to conduct scientific research in new areas using suitable methods, as well as their skills needed for successful research using scientific methods.

**Table 6:** The final index system

First level	Second level	Initial weight	Combined weight	Third level	Initial weight	Combined weight
1. Research literacy (0.5000)	1.1 Basic qualifications	0.2500	0.1250	1.1.1 Language competence	0.5035	0.0629
				1.1.2 Scheduling ability	0.4965	0.0621
	1.2 Research attitude	0.7500	0.3750	1.2.1 Research ethics	0.3348	0.1255
				1.2.2 Research integrity	0.3433	0.1288
				1.2.3 Scientific spirit	0.3219	0.1207
2. Research ability (0.5000)	2.1 Ability to identify problems	0.4076	0.2038	2.1.2 Information capture ability	0.2011	0.0410
				2.1.3 Ability to ask scientific research questions	0.1984	0.0404
				2.1.4 Critical thinking ability	0.2011	0.0410
				2.1.5 Innovative sensitivity	0.2064	0.0421
				2.1.6 Problem transformation ability	0.1930	0.0393
	2.2 Ability to use literature	0.0362	0.0181	2.2.1 Literature retrieval ability	0.2482	0.0045
				2.2.2 Literature reading ability	0.2589	0.0047
				2.2.3 Literature analysis ability	0.2553	0.0046
				2.2.4 Literature quality evaluation ability	0.2376	0.0043
	2.3 Professional capacity	0.1470	0.0735	2.3.1 Professional basic knowledge	0.1285	0.0094
				2.3.2 Professional technical ability	0.1232	0.0091
				2.3.3 Professional foreign language ability	0.1285	0.0094
				2.3.4 Judgment ability	0.1285	0.0094
				2.3.5 Research environment (platform) cognitive ability	0.1180	0.0087
				2.3.6 Interdisciplinary cooperation ability	0.1232	0.0091
				2.3.7 Professional team coordination ability	0.1285	0.0094
				2.3.8 Ability to seek scientific research guidance actively	0.1215	0.0089
	2.4 Subject implementation capacity	0.1686	0.0843	2.4.1 Feasibility analysis ability	0.2517	0.0212
				2.4.2 Subject design ability	0.2517	0.0212
				2.4.3 Subject application writing ability	0.2551	0.0215
				2.4.4 Subject evaluation ability	0.2415	0.0204
	2.5 Data-processing capacity	0.0478	0.0239	2.5.1 Clinical resource collection ability	0.2093	0.0050
				2.5.2 Database usage ability	0.2064	0.0049
				2.5.3 Select a suitable statistical method	0.2064	0.0049
				2.5.4 Statistical software usage ability	0.1802	0.0043
				2.5.5 Qualitative research data analysis and arrangement ability	0.1977	0.0047
	2.6Thesis-writing skills	0.0434	0.0217	2.6.1 Master the writing principles, formats and skills of papers, research reports and declarations	0.3491	0.0076
				2.6.2 Selection of appropriate periodical ability	0.3302	0.0072
				2.6.3 Master the writing of cover letter and reply to reviewers' comments	0.3208	0.0070
	2.7 Research output capacity	0.1494	0.0747	2.7.1 Patent application ability	0.2391	0.0179
				2.7.2 Application for scientific research award-winning ability	0.2428	0.0181
				2.7.3 Paper and monograph publishing ability	0.2609	0.0195
				2.7.4 Transformation ability of approved patents	0.2572	0.0192

## Discussion

In this study, the Delphi method was employed, and after two rounds of expert consultation, in accordance with the characteristics and scientific research requirements of medical staff in China, an index system for the scientific literacy of medical staff in China was constructed. The index system for medical staff's scientific literacy in this study consists of 2 first-level indicators, 9 second-level indicators, and 38 third-level indicators. Medical institutions at all levels can use this index system to scientifically assess medical staff's scientific literacy.

In 2014, the Joint Task Force for Clinical Trial Competency (JTF) published its Core Competency Framework [27]. The Framework focuses more on the capacity to conduct clinical research. These include principles such as clinical research and quality practices for drug clinical trials. However, this framework does not apply to the current evaluation of scientific literacy in hospitals. Because these indicators do not apply to all staff members, there is a lack of practical scientific research, such as information about the final paper output. Therefore, the experts who constructed the index system in this study came from different specialties, and the indicators can be better applied to scientific researchers in all fields. This approach not only addresses clinical researchers but also addresses the concerns of hospital managers, and the indicators are more applicable.

The weighted analysis showed that the primary indicators "research literacy" and "research ability" had the same weight (0.50) and were two important components of scientific literacy. Research ability is a direct reflection of scientific literacy and includes the ability to identify problems, the ability to use literature, professional capacity, subject implementation capacity, data-processing capacity, thesis-writing skills, and research output capacity. Only by mastering these skills can medical staff carry out scientific research activities more efficiently and smoothly. The ability to identify problems refers to the ability of medical staff to obtain insights into the frontiers of their discipline and to identify and ask insightful questions. Ratten claimed that only with keen insight and sufficient sensitivity to major scientific issues can we exploit the opportunities for innovation that may lead to breakthroughs [28]. Therefore, it is suggested that in the process of cultivating the scientific literacy of medical staff, the ability to identify problems, including divergent thinking, innovative sensitivity, and the ability to produce various solutions, should be improved. Furthermore, this study included three subentries of the secondary indicator "research attitude", namely, research ethics, research integrity, and scientific spirit. This is likely because improper scientific research behavior is still prevalent. A study conducted in the United States and Europe showed that the rate of scientific research misconduct was 2% [13]. A small survey conducted in Indian medical schools and hospitals revealed that 57% of the respondents knew that someone had modified or fabricated data for publication [28]. The weight of this index ranked first in the secondary indicators, indicating that scientific attitude is an important condition for improving research quality, relevance, and reliability. Countries and hospitals should develop, implement, and optimize policies and disciplinary measures to combat academic misconduct.

In addition, the third-level indicator "scheduling ability" under the second-level indicator "basic qualification" has a high weight, indicating that medical staff attach importance to management and distribution ability in the context of scientific research. Currently, hospitals face several problems, such as a shortage of medical personnel, excessive workload, and an increase in the number of management-related documents [29, 30]. These factors result in time conflicts between daily responsibilities and scientific research tasks, thereby presenting significant obstacles to the allocation of sufficient time for scientific inquiry [31]. Effectively arranging clinical work and scientific research time is crucial to improving the overall efficiency of scientific research. In the earlier expert interviews, most medical staff believed that scientific research work must be combined with clinical work rather than focused only on scientific research. Having the ability to make overall arrangements is essential to solving these problems. The high weight given to the second-level index of 'subject implementation capacity', along with its associated third-level indicators, highlights the challenges faced by young medical staff in obtaining research subjects. Before implementing a project, researchers must thoroughly investigate, analyze, and compare various aspects of the research project, including its technical, economic, and engineering aspects. Moreover, potential financial and economic benefits, as well as social impacts, need to be predicted to determine the feasibility of the project and develop a research plan [32]. However, for most young medical staff in medical institutions, executing such a project can be challenging due to their limited scientific research experience [33]. A researcher who possesses these skills can truly carry out independent scientific research.

The weights of the second-level index "research output capacity" cannot be ignored. In Chinese hospitals, the ability to produce scientific research output plays a certain role in employees’ ability to obtain rewards such as high pay, and this ability is also used as a reference for performance appraisals [34]. The general scientific research performance evaluation includes the number of projects, scientific papers and monographs, scientific and technological achievements, and patents. In particular, the publication of papers is viewed as an indispensable aspect of performance appraisal by Chinese hospitals [35]. Specifically, scientific research papers are the carriers of scientific research achievements and academic research and thus constitute an important symbol of the level of medical development exhibited by medical research institutions; they are thus used as recognized and important indicators of scientific research output [36]. This situation is consistent with the weight evaluation results revealed by this study.

The results of this study are important for the training and management of the scientific research ability of medical personnel. First, the index system focuses not only on external characteristics such as scientific knowledge and skills but also on internal characteristics such as individual traits, motivation, and attitudes. Therefore, when building a research team and selecting and employing researchers, hospital managers can use the index system to comprehensively and systematically evaluate the situation of researchers, which is helpful for optimizing the allocation of a research team, learning from each other's strengths, and strengthening the strength of the research team. Second, this study integrates the content of existing research to obtain useful information through in-depth interviews with medical staff and constructs an evaluation index system based on Delphi expert consultation science, which comprehensively includes the evaluation of the whole process of scientific research activities. These findings can provide a basis for medical institutions to formulate scientific research training programs, help medical personnel master and improve scientific research knowledge and skills, and improve their working ability and quality. Moreover, the effectiveness of the training can also be evaluated according to the system.

In China, with the emergence of STEM rankings, hospitals pay more and more attention to the scientific research performance of medical personnel. Scientific literacy not only covers the abilities of medical personnel engaged in scientific research, but also reflects their professional quality in this field. Having high quality medical personnel often means that they have excellent scientific research ability, and their scientific research performance will naturally rise. In view of this,,medical institutions can define the meaning of third-level indicators and create Likert scales to survey medical staff. Based on the weights assigned to each indicator, comprehensive scores can be calculated to evaluate the level of scientific literacy among medical staff. Through detailed data analysis, they can not only reveal their shortcomings in scientific research ability and quality, but also provide a strong basis for subsequent training and promotion. Through targeted inspection, we can not only promote the comprehensive improvement of the ability of medical staff, but also promote the steady improvement of their scientific research performance, and inject new vitality into the scientific research cause of hospitals.

### Limitations

This study has several limitations that need to be considered. First, the participants were only recruited from Beijing (a city in China), potentially lacking geographical diversity. We plan to select more outstanding experts from across the country to participate. Second, the index system may be more suitable for countries with medical systems similar to those of China. When applying this system in other countries, some modifications may be necessary based on the local context. Last, While this study has employed scientific methods to establish the indicator system, the index system has yet to be implemented on a large sample of medical staff. Therefore, the reliability and validity of the index system must be confirmed through further research. In conclusion, it is crucial to conduct further detailed exploration of the effectiveness and practical application of the index system in the future.

## Conclusion

This study developed an evaluation index system using the Delphi method to assess the scientific literacy of medical staff in China. The system comprises two primary indicators, nine secondary indicators, and thirty-eight third-level indicators, with each index assigned a specific weight. The index system emphasizes the importance of both attitudes and abilities in the scientific research process for medical staff and incorporates more comprehensive evaluation indicators. In the current era of medical innovation, enhancing the scientific literacy of medical staff is crucial for enhancing the competitiveness of individuals, hospitals, and overall medical services in society. This evaluation index system is universally applicable and beneficial for countries with healthcare systems similar to those of China. This study can serve as a valuable reference for cultivating highly qualified and capable research personnel and enhancing the competitiveness of medical research.

### Supplementary Information

**Supplementary Material 1.**

**Supplementary Material 2.**

**Supplementary Material 3.**

## Data Availability

The datasets used and/or analysed during the current study available from the corresponding author on reasonable request.
