# Supplementary material for: Development of an index system for the scientific literacy of medical staff: a modified Delphi study in China
Source: BMC Med Educ. 2024 Apr 10;24:397. doi: 10.1186/s12909-024-05350-0 (PMC11008007; doi:10.1186/s12909-024-05350-0)
Supplement: Supplementary file 1 — Supplementary Material 1. [file 12909_2024_5350_MOESM1_ESM.pdf]

Supplemental Appendix1

Evaluation index system of medical staff's scientific literacy under the background of high-quality development of hospitals

Expert consultation questionnaire (first round)

Dear Expert,

Thank you for taking part in expert consultation in your busy schedule! In order to make you know more about the purpose and content of this questionnaire, please read the background information of the questionnaire and fill in the informed consent form first.

High-quality development is the theme of the reform and development of public hospitals at present. The scientific literacy of medical staff determines the level of hospital technological innovation and is one of the key factors for hospitals to have core competitiveness. **Scientific literacy** refers to the basic requirements that researchers should meet in terms of ideology and morality, theoretical knowledge and scientific research ability when conducting scientific research. It is an advanced and complex comprehensive quality. Therefore, the purpose of this questionnaire survey is to build an evaluation index system of medical staff's scientific literacy under the background of high-quality development of hospitals, and to guide hospitals to continuously improve their scientific literacy level. The research group initially established an evaluation **index system of medical staff's scientific literacy** under the background of high-quality development of hospitals, including **2 first-level indicators, 10 second-level indicators and 47 third-level indicators.**

In view of your academic achievements and authority in the industry, we sincerely invite you to be an expert in this expert consultation! Your selection and grading are only used for research, and the questionnaire you fill in will be kept strictly confidential. If you have any questions about the questionnaire, please call or send an E-mail for consultation. This survey is expected to require two rounds of questionnaire consultation. At any time during the two rounds of questionnaire survey, you can withdraw from answering the questionnaire without any reason. If you agree to participate in this expert consultation, please sign in the space below. Thank you for your support in your busy schedule!

Other matters:

1. In order to thank you for your support of this study, after two rounds of questionnaire survey, the research group will pay you an expert consultation fee of 1,000 yuan. Please fill in the information table of expert consultation fee below.

2. Please send the questionnaire to this email address or contact person: 2672167681@qq.com within 1-2 weeks.

Contact: Zhai Ziyan 18810550613

Research group on scientific research literacy of medical staff

2022.11

Part I: Basic information of consulting experts (please fill in the serial number or content on the corresponding "___").

1. Name:_______
2. Gender:_______
3. Age(year):_______A ≦30 B 31-40 C 41-50 D 51-60 E ≥60
4. Employer (please fill in the full name) :_______
5. Your occupation:_______

A physician B nurse C full-time researcher D both doctors and hospital administrators E others

6. Your education level:_______

A Bachelor degree or below B Master Degree Candidate C doctoral candidate

7. Your title:_______

A primary B intermediate C sub-senior D senior

8. What are your major fields of expertise at present?

A Basic medicine B clinical medicine C Nursing D Hospital management E others

9. Your current working years in this major (year):_______

A ≦10 B 11-15 C 16-20 D 21-25 E 26-30 F ≥30

Part II: Description of index selection criteria.

1. The importance of indicators: In the evaluation index system, the importance and representativeness of the index. The more important an indicator is, the better its representativeness will be. It can better reflect the connotation of human resource management ability, and the more important the indicator will be. 1 is the least important, 10 is the most important;

2. Feasibility of indicators: In the actual evaluation work, the degree of difficulty to obtain the index. The easier the index is to obtain, the higher the feasibility of the index is. If the indicator data is difficult to obtain, or reliable data is difficult to obtain, or the data obtained is difficult to ensure reliability, or requires a large number of people and money, the feasibility of the indicator will be lower. 1 is the least feasible, 10 is the most feasible;

3. Sensitivity of indicators: In the actual evaluation work, the index has a better ability (sensitivity) to distinguish between vertical and horizontal changes. Vertical change refers to the change between the same region or the same institution at different times; Horizontal change is change between different places or institutions at the same time. Ten scores ranging from 1-10 points were selected, and 1-10 points indicated that the sensitivity of the index was enhanced successively.

4. Your judgment of indicators is affected by four factors: "1 theoretical analysis, 2 practical experience, 3 knowledge from domestic and foreign counterparts, 4 intuition ". The degree of influence is divided into three levels: small (1 point), medium (2 points) and large (3 points). Please select the corresponding degree of influence after the indicator according to your judgment basis of each indicator.

5. Your understanding of the indicators is divided into "5 very familiar, 4 familiar, 3 normal, 2 not familiar, 1 very unfamiliar". Please fill in the corresponding serial number in the table according to the actual situation.

Table 2 Third-level index system evaluation table

First-level	Second-level	Third-level	Index interpretation and calculation formula	Importance (1-10)	Feasibility (1-10)	Sensitivity (1-10)	Modify the opinion (delete, merge, restate)
Research Literacy	Basic Qualification	Language competence	Express your thoughts, feelings, thoughts and intentions clearly and clearly with words, figures, expressions and actions, and be good at letting others understand, experience and master them.				
		Communication ablility	Ability to communicate effectively with others, including external skills and internal motivation.				
		Scheduling ability	Can arrange clinical work and scientific research time effectively.				
		Team leadership ability	Good at scientific assignment of research tasks, and can actively impart business knowledge and skills and guide subordinates to complete tasks.				
		Research talent	Having a natural ability to be good at scientific research or a natural obsession (great enthusiasm) enables them to grow up faster than others with the same experience or even no experience.				
	research morality	Social ethics	Have noble socialist moral quality and social responsibility.				
		Research ethics	Ethical norms and codes of conduct between researchers and collaborators, subjects and ecological environment				
		Research integrity	Adhere to the truth, strictly abide by honesty and trustworthiness, and have good academic ethics.				
	Scientific Attitude	Scientific research motivation	Can be satisfied from scientific research, so scientific research itself has become the driving force for medical personnel to engage in scientific research.				
		Research interest	Be interested in scientific research activities				
		Professional ethics	The degree of attention and seriousness to work, loyalty, dedication, responsibility, initiative, diligence, good at learning, etc.				
		Scientific spirit	Love science and carry out scientific research with a scientific, rigorous, realistic and responsible attitude.				

Research Ability	Ability to identify problems	Information capture ability	It can be filtered through clinical work, literature reading and other channels to obtain valuable information.				
		Ability to ask scientific research questions	Through listening to academic lectures, holding group seminars in the laboratory, reading literature and other ways to grasp the research hotspots, find out clinical disputes, form scientific research ideas, and find existing unsolved problems.				
		Critical thinking ability	Have the courage to break through traditional thinking and inherent thinking, be able to revolutionize and improve, see the shortcomings and defects of others, and be able to evaluate and analyze specific work from a global perspective.				
		Innovative sensitivity	Be sensitive to major scientific issues, and grasp innovation opportunities. grasp the latest medical frontier knowledge in time and apply cutting-edge technology, adapt to the needs of society, and strive to learn new knowledge; Establish a pioneering and enterprising concept suitable for the all-round progress of society				
		Frontier scientific insight.	Have the ability to grasp the latest medical frontier knowledge in time and apply cutting-edge technology, adapt to the needs of society, keep pace with the times and strive to learn new knowledge; Establish a pioneering and enterprising new concept suitable for the all-round progress of society				
		Problem transformation ability	Can transform the unsolved problems into problems that can be solved within the scope of existing knowledge.				
	Ability to use literature	Literature retrieval ability	The process of obtaining documents by using retrieval tools according to the needs of study and work.				
		Literature reading ability	Ability to understand and read documents, including concept understanding, theory understanding, method understanding, viewpoint understanding, document reading skills, logical thinking, mastery of document reading methods, and critical understanding of documents.				
		Literature analysis ability	Ability to analyze the basic composition, organizational structure and logical relationship of documents.				

research ability	Ability to use literature	Document management ability	It involves the collection, analysis, classification and filing of documents and materials.				
		Literature quality evaluation ability	Scientific and effective technical means and evaluation methods (such as Meta-analysis, etc.) are adopted to test its quality and provide evidence of authenticity and reliability.				
	Professional capacity	Professional basic knowledge	Familiarity and mastery of relevant clinical knowledge				
		Professional technical ability	Apply theoretical knowledge to practice, turn knowledge into skills, and master clinical skills skillfully.				
		Professional foreign language ability	Strong foreign language ability, able to read foreign literature and write foreign language articles.				
		judgment ability	In the face of scientific difficulties, we can try a lot and try to do a lot of research to show the research results and make a correct judgment on the problem				
		Access to resources	Be able to use information channels such as institutions, books and periodicals or websites to obtain information.				
		Research environment (platform) cognitive ability	Can clearly understand the ability of scientific research platform in the environment.				
		Interdisciplinary cooperation ability	Use two or more disciplines or professional knowledge systems to provide information, data, technology and theory, and solve problems that can be solved beyond a single scope through mutual integration and understanding.				
		Professional team coordination ability	Cooperate with the team to allocate and mobilize available resources in a timely, reasonable and efficient manner.				

research ability	Subject implementation capacity	Actively seek scientific research guidance ability	There are professionals to assist and guide in scientific research.				
		Feasibility analysis ability	Through the investigation, analysis and comparison of the main contents and supporting conditions of scientific research projects from the aspects of technology, economy and engineering, and the prediction of the possible financial, economic benefits and social impacts after the completion of the project, it is proposed whether the project is worth investing and how to build it.				
		Subject design ability	Scientifically define research types, objectives and methods, ensure internal and external validity of research, put forward research hypotheses, master quality control methods, have the ability to adjust experimental schemes, select research objects, define research variables, determine research methods, and finally form the ability of research schemes.				
		Database usage ability	On the basis of mastering the basic knowledge of database, look for public databases at home and abroad (such as Charls data) and transform the collected database data resources into the capabilities used in their own fields.				
	Data-processing ability	Database organization ability	Be able to check, classify and encode the databases collected in research activities such as investigation, observation and experiment, etc.				
		Select a suitable statistical method	Master the basic concepts of statistics, understand the common data description methods and data analysis methods, and choose the appropriate analysis model.				
		Statistical software usage ability	Master the operation of statistical software				

research ability	Data-processing ability	Qualitative research data analysis and arrangement ability	Ability to effectively describe, synthesize, summarize and summarize qualitative research data.				
	Thesis-writing skills	Master the writing principles, formats and skills of papers, research reports and declarations	Familiar with the writing format, principles, methods and skills of papers.				
		master the writing skills of papers, research reports and declarations.	Familiar with the methods and skills of thesis writing.				
		Familiar with the process of paper submission	Have a certain understanding of the process of publishing academic papers and be familiar with the process of submitting papers.				
		Selection of appropriate periodical ability	According to the major, field, level and orientation of the research, we can find different academic journals of science and technology, and choose the appropriate journals to contribute according to the orientation, characteristics and design columns of the journals.				
	Research output capacity	Patent application ability	Difficulty, category, quantity and level of patents obtained, etc.				

research ability	Research output capacity	Application for scientific research award-winning ability	Factors such as the number, level and ranking of scientific research awards.				
		Paper and monograph publishing ability	The publication quality, quantity, popularity and influence of papers and monographs.				

Please continue to fill in the following questionnaire.

Comments on modification: If you have added, modified or deleted indicators or other suggestions or comments, please briefly explain the reasons and rate the added or modified indicators in the following three aspects: importance (1-5), operability (1-5) and sensitivity (1-5).

Please continue to fill in the following questionnaire.

Table 4 Second-level index system evaluation table

Second-level	Index interpretation	Familiarity (1-5)	Influence degree of the following judgment basis (small 1, medium 2, large 3)				Modify the opinion (delete, merge, restate)
			Theoretical analysis	work experience	Domestic and foreign peers understand	intuition	
Basic Qualification	The ability or obsession (enthusiasm) of medical staff in the field of scientific research, as well as the ability of language communication, management and distribution in scientific research work.						
Research ethics	Medical staff should follow the basic moral standards in their professional activities when they are engaged in scientific and technical work.						
Science attitude	Behavioral tendency, ideological tendency and emotional reaction of medical staff in scientific research activities.						
Ability to identify problems	The ability of medical staff to have insight into the frontier of the discipline, to find and ask questions keenly, and to solve them by themselves.						
Ability to use literature	Medical staff can effectively retrieve and read the literature, manage and analyze the literature and evaluate the quality of the literature.						
Professional capacity	Medical staff's mastery of professional knowledge and technology, cognitive judgment ability, and the ability to mobilize resources through collaboration to fill the shortcomings of different professions.						
Subject implementation capacity	The ability of medical staff to organize and implement a reasonable, feasible and beneficial scientific research project and topic.						
Data-processing capacity	Medical staff have the ability to know, collect, organize, express and explore data.						
Thesis-writing skills	Medical staff have a clear understanding of writing principles, formats, writing skills, and the ability to select journals and contribute.						
Scientific research output capacity	Medical staff can obtain various forms of creative achievements with academic significance or use value through scientific research activities.						

Please continue to fill in the following questionnaire.

Suggestions for revision: If you have any suggestions or opinions on adding, modifying or deleting indicators, please briefly explain the reasons and discuss them from the following eight aspects: importance (1-10), operability (1-10), sensitivity (1-10), familiarity with the indicators (1-5), theoretical analysis (1-3) and practice.

Please continue to fill in the following questionnaire.

Table 5　First-level index system evaluation table

First-level	Index interpretation	Familiarity (1-5)	Influence degree of the following judgment basis (small 1, medium 2, large 3)				Modify the opinion (delete, merge, restate)
			Theoretical analysis	work experience	Domestic and foreign peers understand	intuition	
Research literacy	Including basic literacy, scientific research ethics and scientific research attitude, embodies the essence and core quality of medical staff's scientific and technological innovation, which depend on each other, promote each other and restrict each other, thus promoting the production and application of scientific research results in practice.						
Research Ability	The ability of medical personnel in various fields to conduct scientific research in unknown areas of interest by using appropriate methods or means in scientific thinking and professional activities also refers to the ability of objective skills that scientific researchers need to succeed in scientific research activities by using scientific methods.						

Please continue to fill in the following questionnaire.

Suggestions for revision: If you have any suggestions or opinions on adding, modifying or deleting indicators, please briefly explain the reasons and discuss them from the following eight aspects: importance (1-10), operability (1-10), sensitivity (1-10), familiarity with the indicators (1-5), theoretical analysis (1-3) and practice.
