# Supplementary material for: Development of an index system for the scientific literacy of medical staff: a modified Delphi study in China
Source: BMC Med Educ. 2024 Apr 10;24:397. doi: 10.1186/s12909-024-05350-0 (PMC11008007; doi:10.1186/s12909-024-05350-0)
Supplement: Supplementary file 2 — Supplementary Material 2. [file 12909_2024_5350_MOESM2_ESM.pdf]

Supplemental Appendix2

Evaluation index system of medical staff's scientific literacy under the background of high-quality development of hospitals

Expert consultation questionnaire (second round)

Dear Experts:

Hello! Thank you for taking part in expert consultation in your busy schedule! The first round of consultation letter has been received. Thank you very much for your guidance and help in the first round of expert consultation. According to the principle of scientific and operability of the index system, we adopted your valuable opinions in the first round of consultation. After sorting out and summarizing the opinions put forward by experts in the first round, according to the results of statistical analysis, we revised the expression of the three-level indicators, **deleted nine three-level indicators and added five three-level indicators**. The deleted indicators have been marked with horizontal lines and the added indicators have been marked with italics. In the second round of questionnaire, there are **2 first-level indicators, 10 second-level indicators and 43 third-level indicators**, which need you to evaluate again and further determine the weight of each indicator. Please help and support me again in your busy schedule!

Your selection and grading are only used for research, and the questionnaire you fill in will be kept strictly confidential. If you have any questions about the questionnaire, please call or send an E-mail for consultation. **At any time during the two rounds of questionnaire survey, you can quit the questionnaire for no reason**. Thank you for your support in your busy schedule!

Other matters:

Please send the questionnaire to this email address or contact person: 2672167681@qq.com within 1 week.

Contact: Zhai Ziyan 18810550613

Research group on scientific research literacy of medical staff

2022.11

Part I: Basic information of consulting experts (please fill in the serial number or content on the corresponding "_").

1. Name:

2. Employer (please fill in the full name) :

Part II: Description of index selection criteria.

1． The importance of indicators: In the evaluation index system, the importance and representativeness of the index. The more important an indicator is, the better its representativeness will be. It can better reflect the connotation of human resource management ability, and the more important the indicator will be. <u>1 is the least important, 10 is the most important;</u>

2． Feasibility of indicators: In the actual evaluation work, the degree of difficulty to obtain the index. The easier the index is to obtain, the higher the feasibility of the index is. If the indicator data is difficult to obtain, or reliable data is difficult to obtain, or the data obtained is difficult to ensure reliability, or requires a large number of people and money, the feasibility of the indicator will be lower. <u>1 is the least feasible, 10 is the most feasible;</u>

3． Sensitivity of indicators: In the actual evaluation work, the index has a better ability (sensitivity) to distinguish between vertical and horizontal changes. Vertical change refers to the change between the same region or the same institution at different times; Horizontal change is change between different places or institutions at the same time. Ten scores ranging from 1-10 points were selected, and 1-10 points indicated that the sensitivity of the index was enhanced successively.

4. Your judgment of indicators is affected by four factors: "1 theoretical analysis, 2 practical experience, 3 knowledge from domestic and foreign counterparts, 4 intuition ". The degree of influence is divided into three levels: small (1 point), medium (2 points) and large (3 points). Please select the corresponding degree of influence after the indicator according to your judgment basis of each indicator.

5. Your understanding of the indicators is divided into "5 very familiar, 4 familiar, 3 normal, 2 not familiar, 1 very unfamiliar". Please fill in the corresponding serial number in the table according to the actual situation.

Table 2 Third-level index system evaluation table

First-level	Second-level	Third-level	Index interpretation and calculation formula	Importance (1-10)	Feasibility (1-10)	Sensitivity (1-10)	Modify the opinion (delete, merge, restate)
Research Literacy	Basic Qualification	Language competence	Express your thoughts, feelings, thoughts and intentions clearly and clearly with words, figures, expressions and actions, and be good at letting others understand, experience and master them.				
		~~Communication ablility~~	~~Ability to communicate effectively with others, including external skills and internal motivation.~~	-	-	-	According to the results of the first round questionnaire, this indicator has been deleted.
		Scheduling ability	Can arrange clinical work and scientific research time effectively.				
		Team leadership ability	Good at scientific assignment of research tasks, and can actively impart business knowledge and skills and guide subordinates to complete tasks.				
		~~Research talent~~	~~Having a natural ability to be good at scientific research or a natural obsession (great enthusiasm) enables them to grow up faster than others with the same experience or even no experience.~~	-	-	-	According to the results of the first round questionnaire, this indicator has been deleted.
	research morality	~~Social ethics~~	~~Have noble socialist moral quality and social responsibility.~~	-	-	-	According to the results of the first round questionnaire, this indicator has been deleted.
		Research ethics	Ethical norms and codes of conduct between researchers and collaborators, subjects and ecological environment				

Research Literacy	research morality	Research integrity	Adhere to the truth, strictly abide by honesty and trustworthiness, and have good academic ethics.				
	Scientific Attitude	Scientific research motivation	Can be satisfied from scientific research, so scientific research itself has become the driving force for medical personnel to engage in scientific research.				
		~~Research interest~~	~~Be interested in scientific research activities~~	-	-	-	According to the results of the first round of questionnaire, this indicator has been deleted.
		Professional ethics	The degree of attention and seriousness to work, loyalty, dedication, responsibility, initiative, diligence, good at learning, etc.				
		Scientific spirit	Love science and carry out scientific research with a scientific, rigorous, realistic and responsible attitude.				
Research Ability	Ability to identify problems	Information capture ability	It can be filtered through clinical work, literature reading and other channels to obtain valuable information.				
		Ability to ask scientific research questions	Through listening to academic lectures, holding group seminars in the laboratory, reading literature and other ways to grasp the research hotspots, find out clinical disputes, form scientific research ideas, and find existing unsolved problems.				
		Critical thinking ability	Have the courage to break through traditional thinking and inherent thinking, be able to revolutionize and improve, see the shortcomings and defects of others, and be able to evaluate and analyze specific work from a global perspective.				
		Innovative sensitivity	Be sensitive to major scientific issues, and grasp innovation opportunities. grasp the latest medical frontier knowledge in time and apply cutting-edge technology, adapt to the needs of society, and strive to learn new knowledge; Establish a pioneering and enterprising concept suitable for the all-round progress of society				
		~~Frontier scientific insight.~~	~~Have the ability to grasp the latest medical frontier knowledge in time and apply cutting-edge technology, adapt to the needs of society, keep pace with the times and strive to learn new knowledge; Establish a pioneering and enterprising new concept suitable for the all-round progress of society~~	-	-	-	According to the results of the first round of questionnaire, this indicator has been deleted and merged.

Research Ability	Ability to identify problems	Problem transformation ability	Can transform the unsolved problems into problems that can be solved within the scope of existing knowledge.				
	Ability to use literature	Literature retrieval ability	The process of obtaining documents by using retrieval tools according to the needs of study and work.				
		Literature reading ability	Ability to understand and read documents, including concept understanding, theory understanding, method understanding, viewpoint understanding, document reading skills, logical thinking, mastery of document reading methods, and critical understanding of documents.				
		Literature analysis ability	Ability to analyze the basic composition, organizational structure and logical relationship of documents.				
		Document management ability	It involves the collection, analysis, classification and filing of documents and materials.				
		Literature quality evaluation ability	Scientific and effective technical means and evaluation methods (such as Meta-analysis, etc.) are adopted to test its quality and provide evidence of authenticity and reliability.				
	Professional capacity	Professional basic knowledge	Familiarity and mastery of relevant clinical knowledge				
		Professional technical ability	Apply theoretical knowledge to practice, turn knowledge into skills, and master clinical skills skillfully.				
		Professional foreign language ability	Strong foreign language ability, able to read foreign literature and write foreign language articles.				
		judgment ability	In the face of scientific difficulties, we can try a lot and try to do a lot of research to show the research results and make a correct judgment on the problem				
		~~Access to resources~~	~~Be able to use information channels such as institutions, books and periodicals or websites to obtain information.~~	-	-	-	According to the results of the first round of questionnaire, this indicator has been deleted.
		Research environment (platform) cognitive ability	Can clearly understand the ability of scientific research platform in the environment.				

research ability	Professional capacity	Interdisciplinary cooperation ability	Use two or more disciplines or professional knowledge systems to provide information, data, technology and theory, and solve problems that can be solved beyond a single scope through mutual integration and understanding.				
		Professional team coordination ability	Cooperate with the team to allocate and mobilize available resources in a timely, reasonable and efficient manner.				
		Actively seek scientific research guidance ability	There are professionals to assist and guide in scientific research.				
	Subject implementation capacity	Feasibility analysis ability	Through the investigation, analysis and comparison of the main contents and supporting conditions of scientific research projects from the aspects of technology, economy and engineering, and the prediction of the possible financial, economic benefits and social impacts after the completion of the project, it is proposed whether the project is worth investing and how to build it.				
		Subject design ability	Scientifically define research types, objectives and methods, ensure internal and external validity of research, put forward research hypotheses, master quality control methods, have the ability to adjust experimental schemes, select research objects, define research variables, determine research methods, and finally form the ability of research schemes.				
		Subject application writing ability (increased)	Whether it can accurately and rigorously express the project plan and explain the innovation, value and significance of the project.				
		Subject evaluation ability (increased)	Ability to evaluate the innovation and rationality of scientific research design scheme				
	Data-processing capacity	*Clinical resource collection ability(increased)*	Be able to collect information about the diagnosis and treatment of diseases, individual characteristics of patients and medical services.				
		Database usage ability	On the basis of mastering the basic knowledge of database, look for public databases at home and abroad (such as Charls data) and transform the collected database data resources into the capabilities used in their own fields.				
		~~Database construction ability~~	~~The existing data structure can be selected, organized and stored.~~	-	-	-	According to the results of the first round of questionnaire, this indicator has been deleted.

research ability	Data-processing ability	~~Database organization ability~~	~~Be able to check, classify and encode the databases collected in research activities such as investigation, observation and experiment, etc.~~	-	-	-	According to the results of the first round of questionnaire, this indicator has been deleted.
		Select a suitable statistical method	Master the basic concepts of statistics, understand the common data description methods and data analysis methods, and choose the appropriate analysis model.				
		Statistical software usage ability	Master the operation of statistical software				
		Qualitative research data analysis and arrangement ability	Ability to effectively describe, synthesize, summarize and summarize qualitative research data.				
	Thesis-writing skills	Master the writing principles, formats and skills of papers, research reports and declarations	Familiar with the writing format, principles, methods and skills of papers.				
		~~master the writing skills of papers, research reports and declarations.~~	~~Familiar with the methods and skills of thesis writing.~~	-	-	-	According to the results of the first round of questionnaire, this indicator has been deleted and merged.
		Familiar with the process of paper submission	Have a certain understanding of the process of publishing academic papers and be familiar with the process of submitting papers.				
		Selection of appropriate periodical ability	According to the major, field, level and orientation of the research, we can find different academic journals of science and technology, and choose the appropriate journals to contribute according to the orientation, characteristics and design columns of the journals.				

research ability	Research output capacity	*Master the writing of cover letter and reply to reviewers' comments (increased)*	Be able to write submission letters and reply to reviewers' comments, so that the journal can understand the opinions and ideas that the author wants to express.				
		Patent application ability	Difficulty, category, quantity and level of patents obtained, etc.				
		Application for scientific research award-winning ability	Factors such as the number, level and ranking of scientific research awards.				
		Paper and monograph publishing ability	The publication quality, quantity, popularity and influence of papers and monographs.				
		Transformation ability of approved patents (increased)	Transformation after patent approval				

Please continue to fill in the following questionnaire.

Comments on modification: If you have added, modified or deleted indicators or other suggestions or comments, please briefly explain the reasons and rate the added or modified indicators in the following three aspects: importance (1-5), operability (1-5) and sensitivity (1-5).

Please continue to fill in the following questionnaire.

Table 4 Second-level index system evaluation table

Second-level	Index interpretation	Familiarity (1-5)	Influence degree of the following judgment basis (small 1, medium 2, large 3)				Modify the opinion (delete, merge, restate)
			Theoretical analysis	work experience	Domestic and foreign peers understand	intuition	
Basic Qualification	The ability or obsession (enthusiasm) of medical staff in the field of scientific research, as well as the ability of language communication, management and distribution in scientific research work.						
Research ethics	Medical staff should follow the basic moral standards in their professional activities when they are engaged in scientific and technical work.						
Science attitude	Behavioral tendency, ideological tendency and emotional reaction of medical staff in scientific research activities.						
Ability to identify problems	The ability of medical staff to have insight into the frontier of the discipline, to find and ask questions keenly, and to solve them by themselves.						
Ability to use literature	Medical staff can effectively retrieve and read the literature, manage and analyze the literature and evaluate the quality of the literature.						
Professional capacity	Medical staff's mastery of professional knowledge and technology, cognitive judgment ability, and the ability to mobilize resources through collaboration to fill the shortcomings of different professions.						
Subject implementation capacity	The ability of medical staff to organize and implement a reasonable, feasible and beneficial scientific research project and topic.						
Data-processing capacity	Medical staff have the ability to know, collect, organize, express and explore data.						
Thesis-writing skills	Medical staff have a clear understanding of writing principles, formats, writing skills, and the ability to select journals and contribute.						
Scientific research output capacity	Medical staff can obtain various forms of creative achievements with academic significance or use value through scientific research activities.						

Please continue to fill in the following questionnaire.

Suggestions for revision: If you have added, revised or deleted indicators or other suggestions and opinions, please briefly explain the reasons, and from the following eight aspects: importance (1-5), operability (1-5), sensitivity (1-5), familiarity with the indicators (1-5), theoretical analysis (1-3) and practical experience (1-3).

Please continue to fill in the following questionnaire.

Table 5 First-level index system evaluation table

First-level	Index interpretation	Familiarity (1-5)	Influence degree of the following judgment basis (small 1, medium 2, large 3)				Modify the opinion (delete, merge, restate)
			Theoretical analysis	work experience	Domestic and foreign peers understand	intuition	
Research literacy	Including basic literacy, scientific research ethics and scientific research attitude, embodies the essence and core quality of medical staff's scientific and technological innovation, which depend on each other, promote each other and restrict each other, thus promoting the production and application of scientific research results in practice.						
Research Ability	The ability of medical personnel in various fields to conduct scientific research in unknown areas of interest by using appropriate methods or means in scientific thinking and professional activities also refers to the ability of objective skills that scientific researchers need to succeed in scientific research activities by using scientific methods.						

Please continue to fill in the following questionnaire.

Suggestions for revision: If you have added, revised or deleted indicators or other suggestions and opinions, please briefly explain the reasons, and from the following eight aspects: importance (1-5), operability (1-5), sensitivity (1-5), familiarity with the indicators (1-5), theoretical analysis (1-3) and practical experience (1-3).

Please continue to fill in the following questionnaire.

Part III: AHP index weight scoring.

(1) Instructions for filling in indicators

Table 4 Significance of Importance Numbers

**significance degree**	**meaning**	**significance degree**	**meaning**
**one**	Compared with indicator B, indicator A has the same importance;	**one**	Compared with indicator B, indicator A has the same importance;
**three**	Compared with indicator B, indicator A is slightly more important than indicator B;	**1/3**	Compared with indicator B, indicator A is slightly more important than indicator B;
**five**	Compared with indicator B, indicator A is obviously more important than indicator B;	**1/5**	Compared with indicator B, indicator A is obviously more important than indicator B;
**seven**	Compared with indicator B, indicator A is more important than indicator B;	**1/7**	Compared with indicator B, indicator A is more important than indicator B;
**nine**	Compared with indicator B, indicator A is more important than indicator B;	**1/9**	Compared with indicator B, indicator A is more important than indicator B;

Table 5 Fill in an example (the following figures are assumed to understand how to fill in)

	1 is equally important	3 Slightly important	5 is more important	7 is very important	9 is absolutely important	1/3 is a little unimportant.	1/5 is less important.	1/7 is very unimportant.	1/9 is absolutely not important.	other
**Basic literacy and Compared with scientific research ethics**			√							
**Basic literacy and Compared with scientific research attitude**						√				
**Scientific research ethics and Compared with scientific research attitude**						√				

(2) Index scoring

Table 6 Comparison of first-level indicators (this symbol "√" can be copied to the corresponding position)

	**1 is equally important**	**3 Slightly important**	**5 is more important**	**7 is very important**	**9 is absolutely important**	**1/3 is a little unimportant.**	**1/5 is less important.**	**1/7 is very unimportant.**	**1/9 is absolutely not important.**	**other**
**Compared with research literacy, research ability**										

Please continue to fill in the remaining questionnaires below.

Table 7 Comparison of Secondary Indicators- Research Literacy (this symbol "√" can be copied to the corresponding position)

	1 is equally importa nt	3 Slightly importa nt	5 is more importa nt	7 is very importa nt	9 is absolute ly importa nt	1/3 is a little unimpo rtant.	1/5 is less importa nt.	1/7 is very unimpo rtant.	1/9 is absolute ly not importa nt.	other
Basic qualification and Compared with research ethics										
Basic qualification and Compared with Science attitude										
research ethicsand Compared with Science attitude										

Table 8 Comparison of Secondary Indicators- Research Ability (this symbol "√" can be copied to the corresponding position)

	1 is equally importa nt	3 Slightly importa nt	5 is more importa nt	7 is very importa nt	9 is absolute ly importa nt	1/3 is a little unimpo rtant.	1/5 is less importa nt.	1/7 is very unimpo rtant.	1/9 is absolute ly not importa nt.	other
Ability to identify problems and Compared with the ability to use literature										
Ability to identify problemsand Compared with professional capacity										
Ability to identify problems and Compared with the subject implementation capacity										
Ability to identify problems and Compared with data-processing ability										
Ability to identify problems and Compared with the thesis-writing capacity										
Ability to identify problems and Compared with scientific research output capacity										
Ability to use literatureand Compared with professional capacity										

Please continue to fill in the remaining questionnaires below.

Table 8 Comparison of SecondaryIndicators- Research Ability (You can copy this symbol "√" to the corresponding position) (Continued)

	1 is equally important	3 Slightly important	5 is more important	7 is very important	9 is absolutely important	1/3 is a little unimportant.	1/5 is less important.	1/7 is very unimportant.	1/9 is absolutely not important.	other
Ability to use literatureand and Compared with the subject implementation capacity										
Ability to use literature and and Compared with data-processing ability										
Ability to use literature and and Compared with the thesis-writing skills										
Ability to use literature and and Compared with scientific research output capacity										
Professional capacity and Compared with the subject implementation capacity										
Professional capacity and Compared with data-processing ability										
Professional capacity and Compared with the thesis-writing skills										
Professional capacity and Compared with scientific research output capacity										
subject implementation capacity and Compared with data-processing ability										
subject implementation capacity and Compared with the thesis-writing capacity										
subject implementation capacity and Compared with scientific research output capacity										

Table 8 Comparison of SecondaryIndicators- Research Ability (You can copy this symbol "√" to the corresponding position) (Continued)

	1 is equally importa nt	3 Slightly importa nt	5 is more importa nt	7 is very importa nt	9 is absolute ly importa nt	1/3 is a little unimpo rtant.	1/5 is less importa nt.	1/7 is very unimpo rtant.	1/9 is absolute ly not importa nt.	other
Data-processing ability and Compared with the thesis-writing skills										
Data-processing ability and Compared with scientific research output capacity										
Thesis-writing skills and Compared with scientific research output capacity										
