# Supplementary material for: Development of an index system for the scientific literacy of medical staff: a modified Delphi study in China
Source: BMC Med Educ. 2024 Apr 10;24:397. doi: 10.1186/s12909-024-05350-0 (PMC11008007; doi:10.1186/s12909-024-05350-0)
Supplement: Supplementary file 3 — Supplementary Material 3. [file 12909_2024_5350_MOESM3_ESM.pdf]

Additional files 3 Indicators deleted during the two rounds of consultations

Deleted indicators	Description	Importance	Feasibility	Sensitivity
		F/M/CV	F/M/CV	F/M/CV
[a]Communication ability	Ability to communicate effectively with others, including external skills and internal motivation.	**0.25**/8.35/0.16	0.20/**7.10**/**0.33**	**0.10**/**6.85**/0.26
[a]Research talent	In the scientific research field, the natural ability to be skilled at such research or the natural obsession (enthusiasm) that enables the individual to advance more quickly than others at the same level of experience or even with no experience.	**0.25**/**7.95**/**0.26**	**0.10**/**5.90**/**0.43**	**0.05**/**6.45**/**0.34**
[a]Social ethics	Noble socialist moral quality and social responsibility.	0.55/8.85/0.18	0.25/**6.65**/**0.41**	0.25/**6.55**/**0.46**
[a]Research interest	A fondness for scientific research and exhibition of positive emotional reactions.	0.30/8.35/**0.25**	0.25/**6.90**/**0.39**	0.20/**6.85**/**0.39**
[a]Database construction ability	The existing data structure can be selected, organized and stored.	**0.20**/**7.55**/**0.25**	0.20/7.50/0.28	0.35/7.55/**0.34**
[a]Database organization ability	The ability to check, classify and encode the databases collected in research activities such as investigations or observations and experiments.	**0.25**/**7.55**/**0.27**	0.20/7.50/0.28	**0.15**/7.15/0.31
[a]Statistical software usage ability[*]	Mastery of the method of operating statistical software and the ability to use statistical analysis software correctly.	**0.25**/**7.85**/**0.22**	0.15/7.95/0.18	0.20/7.60/0.24
[b]Team leadership ability	Skill at the scientific assignment of research tasks and the ability to impart business knowledge and skills accurately and guide subordinates to complete tasks.	0.55/4.40/**0.17**	**0.20**/**3.50**/**0.31**	**0.20**/**3.60**/0.28
[b]Scientific research motivation	An interest in scientific research activities that is satisfied by scientific research, thus allowing scientific research itself to become the driving force for medical staff.	0.55/4.40/**0.17**	**0.15**/**3.30**/**0.30**	**0.10**/**3.45**/0.26
[b]Professional ethics	The degree of attention and seriousness of the work; loyalty, dedication, responsibility, initiative, diligence, skill at learning, etc.	0.80/4.75/0.12	0.25/**3.55**/**0.31**	**0.15**/**3.40**/0.28
[b]Document management ability	The collection, analysis, classification and filing of documents and materials.	**0.35**/**4.15**/**0.18**	0.45/4.05/0.25	0.30/**3.60**/**0.32**
[b]Familiar with the process of paper submission	The ability to write submission letters and reply to reviewers' comments to ensure that the journal can understand the opinions and ideas that the author wants to express.	0.55/4.30/0.20	0.40/4.10/0.22	0.35/**3.60**/**0.34**

[*]The indicator was retained based on the results of the expert group discussion. [a]represents the first round; [b]represents the second round. Bold numbers are values that do not meet the

threshold criteria. CV, coefficient of variation; F, full score frequency; M, arithmetic mean
